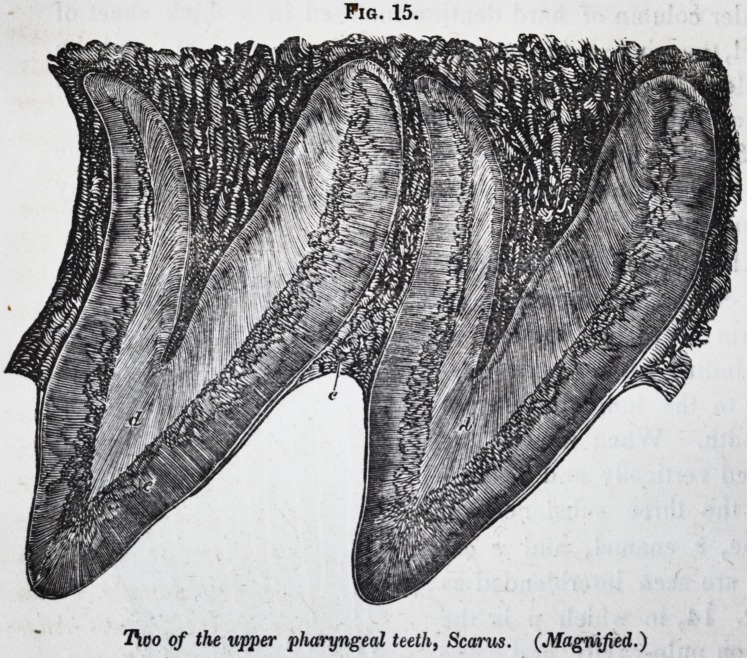# Teeth—Comparative Anatomy

**Published:** 1852-01

**Authors:** 


### SELECTED ARTICLES.

### ARTICLE X.

### *Teeth—Comparative Anatomy.*

[Sing. *a tooth; Tunth,* Teut.; *Dens,* Lat; *Dente,* Ital.; *Dent,* Fr.; *Tand,* Dan.; *Tain,* Old English; *Zahn,* Germ.; *Dant,* Welsh; *Dend,* Erse; *οδους-ιδοντος,* Gr.; *Dantis,* Lithuanic; *Dantas,* Sanscrit.*]

A tooth is a hard body attached to the mouth or commencement of the alimentary canal, always exposed, save where its development is permanently arrested, as in the rudimental tusk of the Narwhal; commonly calcified, the exceptions being few, *e. g.*, the horny teeth of the lamprey and platypus. Teeth vary, not only in their tissue, but still more in number, size, form, structure, position and mode of attachment, in different animals: they are principally adapted for seizing, tearing, dividing, pounding or grinding the food; in some they are modified to serve as weapons of offence and defence; in others, as aids in locomotion, means of anchorage, instruments for uprooting or cutting down trees, or for transport and working of building materials; they are characteristic of age and sex; and in man they have secondary relations, subservient to beauty and to speech.

Teeth are always most intimately related to the food and hab-

*These synonyms are cited as illustrative of the coincidence in one of the primary words of a natural class of languages that prevails from the East Indies, through the west of Asia and across Europe, and as indicative of the unity of stock of the great Indo-European family of mankind.

its of the animal, and are therefore highly interesting to the physiologist: they form, for the same reason, most important guides to the naturalist in the classification of animals; and their value as zoological characters is enhanced by the facility with which, from their position, they can be examined in living or recent animals; whilst the durability of their tissues renders them not less available to the palæontologist in the determination of the nature and affinities of extinct species, of whose organization they are often the sole remains discoverable in the deposits of former periods of the earth's history.

Although there are many analogous structures in the invertebrate classes, true calcified teeth are peculiar to the vertebrata, and may be defined as bodies primarily, if not permanently, distinct from the skeleton, consisting of a cellular and tubular basis of animal matter containing earthy particles, a fluid, and a vascular pulp.

In general, the earth is present in such quantity as to render the tooth harder than bone, in which case the animal basis is gelatinous, as in other hard parts where a great proportion of earth is combined with animal matter. In a very few instances among the vertebrate animals, the hardening material exists in a much smaller proportion, and the animal basis is albuminous; the teeth here agree, in both chemical and physical qualities, with horn.

True teeth consist commonly of two or more tissues, characterised by the proportions of their earthy and animal constituents, and by the size, form, and direction of the cavities in the animal basis, which contains the earth, the fluid, or the vascular pulp.

The tissue which forms the body of the tooth, is called "dentine," (*Dentinum*, Lat.; *Zahnbein*, *Zahnsubstanz*, Germ.; *l' Ivoire*,* Fr.)

The tissue which forms the outer crust of the tooth, is called "cement" (*cæmentum*, *crusta petrosa*, Lat.)

*The learned author of the article "Secretions," in the "Dictionnaire Universel d'Histoire Naturelle," 8vo, 1848, adopts the term "Dentine" in prefer-

The third tissue, when present, is situated between the dentine and cement, and is called "enamel" *encaustum*, *adamas*, Lat.)

"*Dentine*" consists of an organized animal basis disposed in the form of extremely minute tubes and cells, and of earthy particles: these particles have a twofold arrangement, being either blended with the animal matter of the interspaces and parietes of the tubes and cells, or contained in a minutely granular state in their cavities. The density of the dentine arises principally from the proportion of earth in the first of these states of combination, the tubes and cells contain, besides the granular earth, a colorless fluid, probably transuded "plasma" or "liquor sanguinis," and thus relate not only to the mechanical conditions of the tooth, but to the vitality and nutrition of the dentine.

This typical structure of dentine is well illustrated in the article *tooth:* such "true dentine" has no canals large enough to admit capillary vessels with the red particles of blood, and it has been, therefore, called "unvascular dentine."

The simplest modification of dentine is that in which capillary tracts of the primitive vascular pulp remain uncalcified, and permanently carry red blood into the substance of the tissue. These so-called "medullary canals" or "vascular canals" present various dispositions in the dentine which they modify, and which I have proposed to call "vaso-dentine." It is often combined with true dentine in the same tooth; *e. g.* in the scalpriform incisors of certain rodents,* the tusks of the elephant,† the molars of the extinct iguanodon.‡

ence to Cuvier's name "ivoire," and after defining its properties, observes, "Sur ces divers rapports, le mot *dentine*, par lequel M. R. Owen les désigne, me paraît très heureux." The term "ivory" unavoidably recalls the idea of the peculiar modification of "dentine," which characterises the tusks of the elephant, mammoth and mastodon; but, besides this objection to its more general application, the word is used in a still wider sense in the "Leçons d'Anatomie Comparée:" "Les unes (dents) en effet, ont la partie enfoncée dans l'alvéole dénuée d'émail; cette partie, ou la racine, ne se compose généralement que de l'*ivoire* intérieure, recouvert très rarement d'*ivoire* extérieure (les dents de cachalot;") tom. iv. Ed. posth. 1836, p. 200. The example cited of the tissue here denominated "ivoire extérieure," is the "cement." See my "Odontography," p. 355, pl. 89.

* Odontography, 4to, p. 405. † Ib. p. 643. ‡ Ib. p. 251.

A third modification of the fundamental tissue of the tooth is where the cellular basis of the dentine is arranged in concentric layers around the vascular canals, and contains "radiated cells" like those of the osseous tissue: it is called "osteo-dentine." The transition from dentine to vaso-dentine, and from this to osteo-dentine, is gradual, and the resemblance of osteo-dentine to true bone is very close.

"*Cement*" always closely corresponds in texture with the osseous tissue of the same animal; and wherever it occurs of sufficient thickness, as upon the teeth of the horse, sloth, or ruminant, it is also traversed, like bone, by vascular canals. In reptiles and mammals, in which the animal basis of the bones of the skeleton is excavated by minute radiated cells, forming with their contents the "corpuscles of Purkinje," these are likewise present, of similar size and form, in the "cement," and are its chief characteristic as a constituent of the tooth. The hardening material of the cement is partly segregated and combined with the parietes of the radiated cells and canals, and is partly contained in disgregated granules in the cells, which are thus rendered white and opaque, viewed by reflected light. The relative density of the dentine and cement varies according to the proportion of the earthy material, and chiefly of that part which is combined with the animal matter in the walls of the cavities, as compared with the size and number of the cavities themselves. In the complex grinders of the elephant, the masked boar, and the capybara, the cement, which forms nearly half the mass of the tooth, wears down sooner than the dentine.

The "*enamel*" is the hardest constituent of a tooth, and, consequently, the hardest of animal tissues; but it consists, like the other dental substances, of earthy matter arranged by organic forces in an animal matrix. Here, however, the earth is mainly contained in the canals of the animal membrane; and, in mammals and reptiles, completely fills those canals, which are comparatively wide, whilst their parietes are of extreme tenuity. The hardening salts of the enamel are not only present in far greater proportion than in the other dental tissues,

FIG. 1.

*Section of pharyngeal tooth of Labrus, magnified. (Owen's Odontography.)*

FIG. 2.

*Section of tooth of Lamna, magnified. (Owen's Odontography.)*

but, in some animals, are peculiarly distinguished by the presence of fluate of lime.

The following are characteristic examples of the above defined tissues, and their different combinations, in different teeth.

The examples are extremely few, and, as far as I know, are peculiar to the class *pisces*, of calcified teeth which consist of a single tissue, and this is always a modification of dentine. The large pharyngeal teeth of the wrasse *(labrus)* consist of a very hard kind of unvascular dentine. *Fig.* 1, shows a vertical section of one of these teeth, supported upon the very vascular osseous tissue of the pharyngeal bone: *p* is the pulp cavity.

The next stage of complexity is where a portion of the dentine is modified by vascular canals. Teeth, thus composed of dentine and vaso-dentine, are very common in fishes. The hard dentine is always external, and holds the place, and performs the office, of enamel in the teeth of higher animals; but it is only analogous to enamel, not the same tissue.* *Fig.* 2, illustrates this structure in a longitudinal section

* Odontography, pp. 17, 37.

of a tooth of a shark of the genus *lamna: v* is the vaso-dentine; *d* the hard dentine; the earthy constituent so predominates that the tissue takes a polish like enamel, for which it has commonly been mistaken in the teeth of fishes: I have called it "vitro-dentine."

The molars of the dugong are examples of teeth composed of dentine and cement, the latter tissue forming a thick external layer. *Fig.* 3. A is a transverse section of the crown

Fig. 3.

*Section of tooth of Dugong (halicore,) magnified.*

of the second molar, natural size; and B a magnified view of a portion of the section; *d* the dentime, remarkable for the number of minute calcigerous cells at its periphery; and *c* the cement.

In the great teeth of the lower jaw of the cachalot, the pulp-cavity of the growing tooth becomes filled up by osteo-dentine, the result of a modified calcification of the dentinal pulp; and the full grown tooth presents three tissues, as shown in

*fig*. 4, in which *c* is the thick external cement, *d* the hard dentine, and *o* the osteo-dentine; sometimes developed in loose stalactitic-shaped nodules.

In the teeth of the sloth, and its great extinct congener, the megatherium, the hard dentine is reduced to a thin layer, and the chief bulk of the tooth is made up of a central body of vaso-

FIG. 4.

*Section of tooth of Cachalot (physeter.)*

FIG. 5.

*Section of tooth of Megatherium.*

dentine, and a thick external crust of cement. *Fig*. 5 represents a longitudinal section of a lower molar of the megatherium, of half the natural size: *v* is the vaso-dentine, *d* is the hard dentine, and *c* is the cement; *p* is the base of the wide persistent pulp-cavity.

The hard dentine is, of course, the firmest tissue of a tooth so composed, and forms the crest of the transverse ridges of the grinding surface, like the enamel plates in the elephant's grinder. It has, consequently, been described to be enamel,* but its relation to that tissue is only one of analogy or function.

The human teeth, and those of the carnivorous mammals, appear at first sight to be composed of dentine and enamel only, as they were described to be by the Cuviers,† who called them, therefore, simple teeth; but their crowns are originally, and their fangs are always, covered by a thin coat of cement. There is also commonly a small central tract of osteo-dentine in old teeth.

FIG. 6.

*Section of incisor of a Horse (equus.)*

In *fig.* 7, pl. 122, of my Odontography is given a longitudinal section of a human molar tooth, in which *d* is the dentine, *e* the enamel, and *c* the cement.

The teeth, called by Cuvier compound or complex in mammalia, differ, as regards their composition, from the preceding, only by the different proportion and disposition of the constituent tissues. *Fig.* 6, is a longitudinal section of the incisor of a horse; *d* is the dentine, *e* the enamel, and *c* the cement; *c'* is the layer of cement reflected into the deep central depression of the crown; and *s* is the colored mass of tartar and particles of food which fills up that cavity, forming the "mark" of the horse-dealer. The characteristic structure of the three tissues is shown in the magnified part of the section, *fig.* 7.

A very complex tooth may be formed out of two tissues by the way in which these may be interblended, as the result of an

*Cuvier, Ossemens Fossiles, 4to, t. v., pt. 1., p. 172.; and Clift, Transactions of the Geological Society, 1835, p. 438.

†F. Cuvier, Dents de Mammifères, p. 1. 8vo, 1825; G. Cuvier, Leçons d'Anat. Comp. iv, (1836,) p. 199.

original complex disposition of the constituents of the denta matrix.

Certain fishes, and a singular family of gigantic extinct batrachians, which I have called "labyrinthodonts,"* exhibit, as the name implies, a remarkable instance of this kind of complexity. *Fig*. 8, is a view of a canine tooth of the *labyrinthodon salamandroïdes*, of the natural size: and *fig*. 9, is a slightly magnified view of a transverse section across the part of the crown marked *a*. At first view, the tooth appears to be of the simple conical kind, with the exterior surface merely stri-

Fig. 7.

*Magnified portion of section of incisor of Horse ; c* cement, *e* enamel, *d* dentine.

Fig. 8.

*Tooth of a Labyrinthodon, natural size.*

* Proceedings of the Geological Society, Jan. 20, 1841, p. 257.

ated longitudinally, but every streak is a fissure into which the very thin external layer of cement (*c*) is reflected into the body

Fig. 9.

*Transverse section of tooth of Labyrinthodon. (Magnified.)*

of the tooth, following the sinuous wavings of the lobes of dentine *d*, which diverge from the central pulp-cavity, *a*.

The inflected fold of cement *c* runs straight for about half a line, and then becomes wavy, the waves rapidly increasing in breadth as they recede from the periphery of the tooth; the first two, three, or four undulations are simple; then their contour itself becomes broken by smaller or secondary waves; these become stronger as the fold approaches the centre of the tooth, when it increases in thickness, and finally terminates by a slight dilatation or loop close to the pulp-cavity, from which the free margin of the inflected fold of cement is separated by an extremely thin layer of dentine. The number of the inflected converging folds of dentine is about fifty at the middle of the crown of the tooth figured, but is greater at the base. All the inflected folds of cement at the base of the tooth have the same complicated disposition with increased extent; but, as they approach their termination towards the upper part of the tooth, they also gradually diminish in breadth, and consequently penetrate to a less distance into the substance of the tooth.

Hence, in such a section as is delineated (*fig.* 9,) it will be observed that some of the convoluted folds, as those marked *cc*, extend near to the centre of the tooth; others, as those marked *c′*, reach only about half-way to the center; and those folds, *c″*, which, to use a geological expression, are "cropping out," penetrate to a very short distance into the dentine, and resemble, in their extent and simplicity, the converging folds of cement in the fangs of the tooth of the ichthyosnurus.

The disposition of the dentine is still more complicated than that of the cement. It consists of a slender, central, conical column, excavated by a conical pulp-cavity for a certain distance from the base of the tooth; and this column sends radiatting outwards, from its circumference, a series of vertical plates, which divide into two once or twice before they terminate at the periphery of the tooth.

Each of these diverging and dichotomising plates gives off throughout its course smaller processes, which stand at right angles, or nearly so, to the main plate; they are generally opposite, but sometimes alternate; many of the secondary plates or processes, which are given off near the center of the tooth, also divide into two before they terminate; and their contour is seen, in the transverse section, to partake of all the undulations of the folds of cement which invest and divide the dentinal plates and processes from each other.

The dental pulp-cavity is reduced to a mere line, about the upper third of the tooth, but throughout its whole extent fissures radiate from it, corresponding in number with the radiating plates of dentine. Each fissure is continued along the middle of each plate, dividing where this divides, and extending along the middle of each bifurcation and process to within a short distance of the line of cement. The pulp fissure commonly dilates into a canal at the origin of the lateral processes of the radiating plates, before it divides to accompany and penetrate those processes.

The main fissures or radiations of the pulp-cavity extend to within a line or half a line of the periphery of the tooth, and suddenly dilate at their terminations into spaces, which in transverse section, are subcircular, oval, or pyriform, *p* : the branches of the radiating lines, which are continued into the lateral secondary plates or processes of the dentinal lamellæ, likewise dilate into similar, and generally smaller spaces. All these spaces, or canals, in the living tooth, must have been occupied by corresponding processes of the vascular pulp : they constitute so many centers of radiation of the fine calcigerous tubes, which with their uniting clear substance, constitute the dentine.*

Fig. 10

*Tooth of a Dendrodus, natural size.*

An analogous complexity is produced by numerous fissures radiating from a central mass of vaso-dentine, which more or less fills up the pulp-cavity of the seemingly simple conical teeth of the extinct family of fishes which I have called "Dendrodonts."† *Fig.* 10, is one of these fossil teeth, of the natural size ; *a* a transverse section ; and *fig.* 11, a reduced view of a portion of the same section, enlarged twenty diameters.

Thus magnified, a central pulp-cavity, of relatively small size, and of an irregular lobulated form, is discerned, a portion of which is shown at *p* ; this is immediately surrounded by the transverse sections of large cylindrical medullary or pulp canals of different sizes ; and, beyond these, there are smaller and more numerous medullary canals, which are processes of the central pulp-cavity. In the transverse section these processes are seen to be connected together by a net-work of smaller medullary canals belonging to a coarse osseous texture into which the pulp has been converted, and this structure occupies the middle half of the section. All the medullary canals were filled by the opaque matrix. From the circumference of the central net-work, straight medullary fissures radiate at pretty regular intervals to the periphery of the tooth : most of these canals divide once, rarely twice, in their course ; the division taking place sometimes at their origin, in others at different distances

*Odontography, pp. 195—217, pl. 64 A, 64 B.

Fig.. 11

*Transverse section of a tooth of Dendrodus.* *A*, natural size ; *B*, the portion *c*, of *A*, magnified twenty diameters.

from their terminations, and the branches diverge slightly as they proceed. Each of the above medullary fissures is continued from a short process of the central structure, which is connected by a concave line with the adjoining process, so that the whole periphery of the transverse section of the central coarse reticulo-medullary body of the tooth presents a crenate outline. From each ray and its primary dichotomous divisions, short branches are sent off at brief intervals, generally at right angles with the trunk, or slightly inclined towards the periphery of the tooth. These subdivide into a few short ramifications, like the branches of a shrub, and terminate in irregular and somewhat angular dilatations, simulating leaves, but which resolve themselves into radiating fasciculi of calcigerous tubes. There are from fifteen to twenty-five or thirty-six of these short and small lateral branches on each side of the medullary rays.

A third kind of complication is produced by an aggregation of many simple teeth into a single mass.

The examples of these truly compound teeth* are most common in the class of fishes, but the illustration here selected is from the mammalian class. Each tooth of the cape ant-eater (*orycteropus*) presents a simple form, is deeply set in the jaw, but without dividing into fangs; its broad and flat base is porous, like the section of a common cane. The canals to which these pores lead contain processes of a vascular pulp, and are the centers of radiation of as many independent series of dentinal tubules. Each tooth, in fact, consists of a congeries of long and slender prismatic denticles of dentine, which are cemented together by their ossified capsules, the columnar denticles slightly decreasing in diameter and occasionally bifurcating as they approach the grinding surface of the tooth.

Fig. 12.

*Part of transverse section of the tooth of the Orycteropus. (Magnified.)*

A figure of a longitudinal section of the molar teeth is given in pl. 76, *fig.* 10, of my "Odontography," and a magnified view of a similar section in pl. 77; *fig.* 12, gives a magnified view of a portion of the transverse section of the fourth molar, showing *c* the cement; *d* the dentine; *p* the pulp-cavity of the denticles; and *d* a section of one of the denticles just beyond its bifurcation.

The pectinated incisors of the flying lemur of the Indian Islands (*galeopithecus*) are exam-

* "In the "Lecons d'Anatomie Comparée" of Cuvier, the teeth, in which folds of enamel and cement penetrate the entire substance of the crown, are called "compound:" "Nous appellons 'dent composée' celle dont les différentes substances forment des replis tellement profonds, que dans quelque sens qu'on coupe là dent, on coupe plusieurs fois chacune des substances qui la composent: telles sont les dents molaires de l'*Eléphant.*" The teeth of the "Labyrinthodonts" would come under this definition more truly than those of the elephant, although they differ from them in having no enamel; for a molar of an elephant might be bisected, vertically and transversely, without cutting the tissues across more than once.

ples of teeth, the crowns of which are composed of denticles consisting of hard dentine, with a covering of true enamel. The layer of cement over this is too thin to show its characteristic structure, and does not fill up the intervals of the denticles, which stand out as free processes from the base of the crown. Tubular prolongations of the pulp-cavity are continued up the center of each denticle.

*Fig.* 13, exhibits a longitudinal section magnified, of this kind of compound tooth: *d* is the dentine; *e* the enamel; *p*

FIG. 13.

*Section of lower incisor of Galeopithecus. (Magnified.)*

the pulp-cavity. The originally detached summits of the crown of the human incisor are homologous with these columnar processes, or denticles of the incisor of the *galeopithecus.*

In the compound molars of the great African wart-hogs (*phacochœrus*) the columnar denticles are in three rows, and their interspaces are filled up by cement: each denticle consists of a slender column of hard dentine inclosed in a thick sheet of enamel, the whole being bound together by the cement; and the denticles, as in the *galeopithecus*, blending together into a common base in the fully developed tooth.

A figure is given of the grinding surface of the third true molar of the *phacochœrus pallasii*, in pl. 140, *fig*. 4, of my "Odontography."

Fig. 14.

*Section of molar of Elephant.*

In the elephant the denticles of the compound molars are in the form of plates, vertical to the grinding surface and transverse to the long diameter of the tooth. When the tooth is bisected vertically and lengthwise, the three substances, *d* dentine, *e* enamel, and *c* cement, are seen interblended as in *fig*. 14, in which *p* is the common pulp-cavity, and *r* one of the roots of this complex tooth.

A still more complex grinding apparatus is found in certain fishes. The lower pharyngeal bone of the parrot-fish (*scarus**,) for example, supports a dental plate with a triturating surface like that of the compound molars of the *phacochœrus*. The interlocked upper pharyngeals (*fig*. 22,) support dental masses with a grinding surface more like that of the compound molars of the elephant.

When a vertical and longitudinal section is made of one of these upper pharyngeal compound teeth, each denticle is seen to be composed, as in *fig*. 15, of a body of very hard and unvascular dentine *d*, with a thick sheath of enamel *e*, the denticles being united together by the cement *c*, and supported and further united together, and to the pharyngeal bone, by a basal mass of vascular osteo-dentine.

* Odontography, pl. 51, fig. 3.

FIG. 15.

*Two of the upper pharyngeal teeth, Scarus.* (*Magnified.*)

Such are some of the prominent features of a field of observation which comparative anatomy opens out to our view; such the varied nature, and such the gradation of complexity of the dental tissues, which, up to December, 1839,* continued notwithstanding successive approximations to the truth, to be described in systematic works as a "phaneros," or "a dead part or product exhaled from the surface of a formative bulb!" The truth may be slowly, but as surely established, subject to the usual attempts to mask or detract from the merit of the discovery. By no systematic authors has the hypothesis of the formation of dentine by transudation or secretion been more frequently or more explicitly enunciated than by the Cuviers. Baron Cuvier repeats, in both editions of his elaborate work—

* See the Fasciculus of M. de Blainville's great work, "Ostéographie et Odontographie d'Animaux Vertébrés," which he submitted to the Academy of Sciences of the Institute of France on the same day, December 16th, 1839, on which I communicated, on the occasion of my election as corresponding member of that body, my "Theory of the development of dentine by centripetal calcification and conversion of the cells of the pulp."

the "Ossemens Fossiles"—"C'est dans ce vide conçevable que se déposeront les matiéres qui doivent former la dent, savoir : la substance vulgairement appelée *osseuse*, qui sera transudée par des productions gelatineuses venant du fond de la capsule, et l'émail qui sera déposé par les cloisons membraneuses." t. ii, p. 61, ed. 1812; t. i. p. 33, ed. 1821. See also M. F. Cuvier, "Dents de Mammifères," 8vo, 1825. "L'ivoire se dépose par couches concentriques," p. xxvii; "L'émail se dépose dans un sens contraire l'ivoire," ib. p. xxviii. And Baron Cuvier again, in the second edition of his "Leçons d'Anatomie Comparée," t. iv. 1836, p. 213: "L'ivoire se dépose par couches, par une sorte de transudation." In the first edition of his classical work, Cuvier had illustrated the peculiarity of the teeth of certain fishes, which are at first detached and afterwards united to the jaw bone, by comparing their growth to that of the epiphyses of the long bones: "Mais les dents qui ne tiennent qu'à la gencive seulement, comme celle des *Squales*, croissent à la manière des epiphyses des os, c'est-à-dire que toute leur substance osseuse est d'abord tendre et poreuse, et qu'elle se durcit uniformèment, et finit par devenir entiérement dure comme de l'ivoire," t. iii. 1805, p. 112. Whether the great anatomist meant to imply that the osseous tissue of the epiphyses of bones was developed differently from osseous tissue in general, *e. g.* by the uniform and simultaneous hardening or calcification, obscurely referred to in the above quotation, may be questioned, for such is not the way in which the teeth of the shark are calcified. But this is certain, that the idea, whatever it might have been, had no influence on the fixed belief of the development of the dental tissue by transudation expressed in their later and more elaborate works by Baron Cuvier and his accomplished brother; and, in point of fact, the passage which I have quoted is expunged from the second edition of the "Leçons d'Anatomie Comparée," 1835: the successive stages of calcification in the different teeth of the same vertical series in the jaw of the shark, having probably been noticed in the interim by Cuvier.

The author of the article "Sécrétions" in the "Dictionnaire

Universel d'Histoire Naturelle," has, however, reproduced Cuvier's obscure comparison of certain fishes' teeth to the epiphyses of bone, as evidence of the needlessness of any ulterior researches for the demonstration of the theory of dental development by conversion and calcification of the pulp. The passage from the third vol. of the old edition (1800) of the "Leçons d'Anat. Comp." p. 112, is cited to show that it naturally conducts to the knowledge of such mode of development of dentine: "En 1840 et 1841 (the 'Comptes Rendus de l'Acad. des Sciences' give the true date) l'étude des dents de Squale par M. R. Owen, lui a démontrée leur accroissement par intussusception, comme elle avait été à G. Cuvier trente-cinq années auparavant." How or why G. Cuvier came to abandon the theory so demonstrated, and how it happened that none of his contemporaries adopted it, M. Duvernoy does not explain. He does give a reason for the omission, in the second edition of the "Leçons d'Anat. Comp." of the passage which he affirms to contain the demonstration: Malheureusement, le copiste de cet ancien texte pour la 2de édition a omis ce passage, par oubli." It was natural to conclude that its obscurity and seeming contradiction to the theory of dental development, formerly propounded by Cuvier, as well as to the facts shown by nature in the sharks, had been the cause of its omission; but even had the misfortune to which M. Duvernoy now attributes that omission (for in the copious list of addenda and corrigenda to the fifth, 1837, and final, 1846, volumes it is not noticed) not occurred, the coincidence of such passages as the following would still have been inexplicable and irreconcilable with the deductions that M. Dumeril is now enabled to draw from the comparison of the shark's tooth with the epiphyses of long bones. "L'ivoir se dépose par couches, par une sorte de transudation." Leçons d'Anat. Comparée, t. iv., 1836, p. 214. To which proposition Cuvier has himself added a note: "Je me suis assuré récemment, sur des germes de dents d'éléphant, que la substance osseuse de la dent se forme comme les coquilles." And the editor, (M. Duvernoy,) in order to obviate any possibility of misconception, has himself subjoined a note to that passage, as follows: "L' *ivoire* a été

aussi appellé *substance osseuse*, à cause de son analogie de composition chimique et de dureté avec les os. Mais la nature inerte et inorganique de cette substance, mieux appreciée dans ces derniers temps, surtout par les travaux de M. Cuvier, ne permet plus de la désigner, avec justesse, par cette seconde expression. Du moins est-il nécessaire de prémunir le lecteur contre l'ideé fausse qu'il pourrait en tirer, qu'elle serait organisée, qu'elle se développerai, à la manière des os." Tom. cit. p. 201, (1836.) In the same spirit in which M. Duvernoy sees (in 1848) that a true idea, instead of a false one, may be drawn from casual expressions and similies loosely applied in the old Leçons of 1800 and 1805 ; others have sought to depreciate the value of the establishment of the truth by citing the doubts, or tentative approximations made by Purkinje and Schwann to my theory, interpreting such approximations by the light of the established truth. So far from finding such a resting place for doubt in Cuvier's early simile, cited by M. Duvernoy in 1848, or in the interrogatories of Schwann, nothing short of the investigation of the whole of this vast subject, zootomically, developmentally, and microscopically, as narrated in my "Odontography," sufficed to settle my own doubts ; and nothing short of the evidence and illustrations given in that work appeared to me adequate to convert anatomists from the excretion-hypothesis to the intussusception theory.

That the dentine is the ossified pulp is an older notion than that it is an inorganic secretion from such pulp. But an hypothesis, to be of any value in science, must be proved. Almost every true theory has been indicated, with various degrees of approximation, before its final establishment: but he has even been held, in exact philosophy, to be the author of a theory, by whom it has been first rightly enunciated and satisfactorily established. When time has dissipated the mists of individual or national rivalries and jealousies, the name of the true discoverer is clearly seen by the inextinguishable light of true and impartial history: and to that period I look forward with calm and confident hope.

(To be Continued.)

## Figures and Tables

**Fig. 1. f1:**
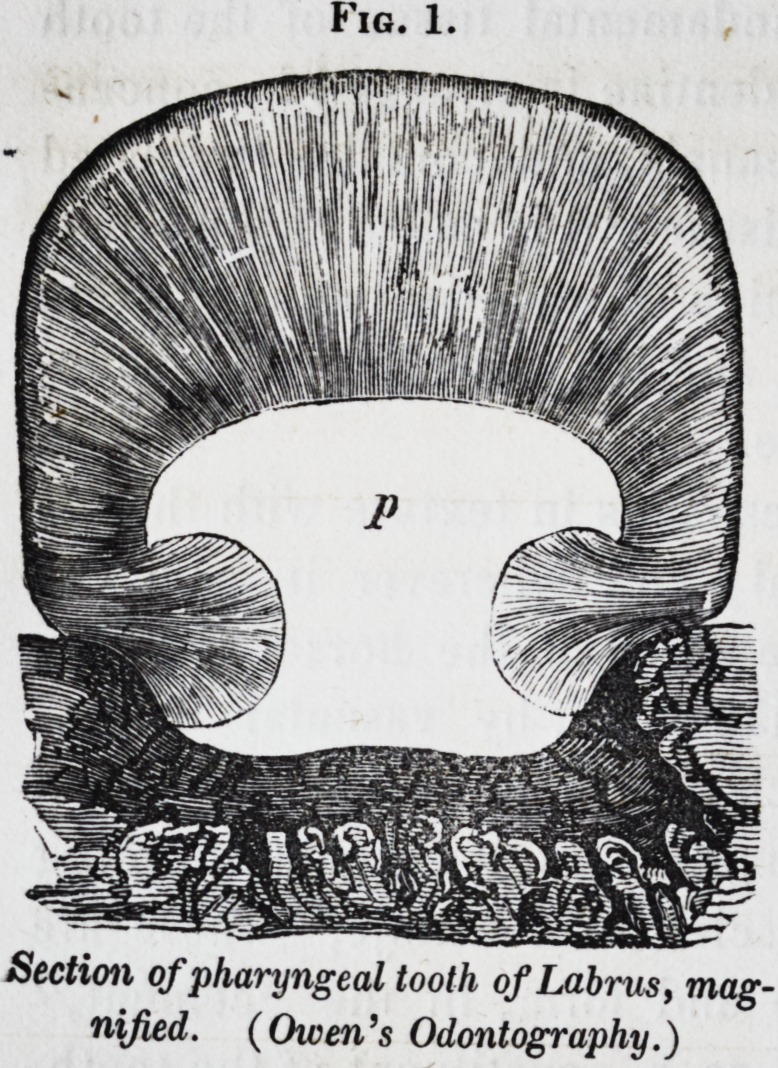


**Fig. 2. f2:**
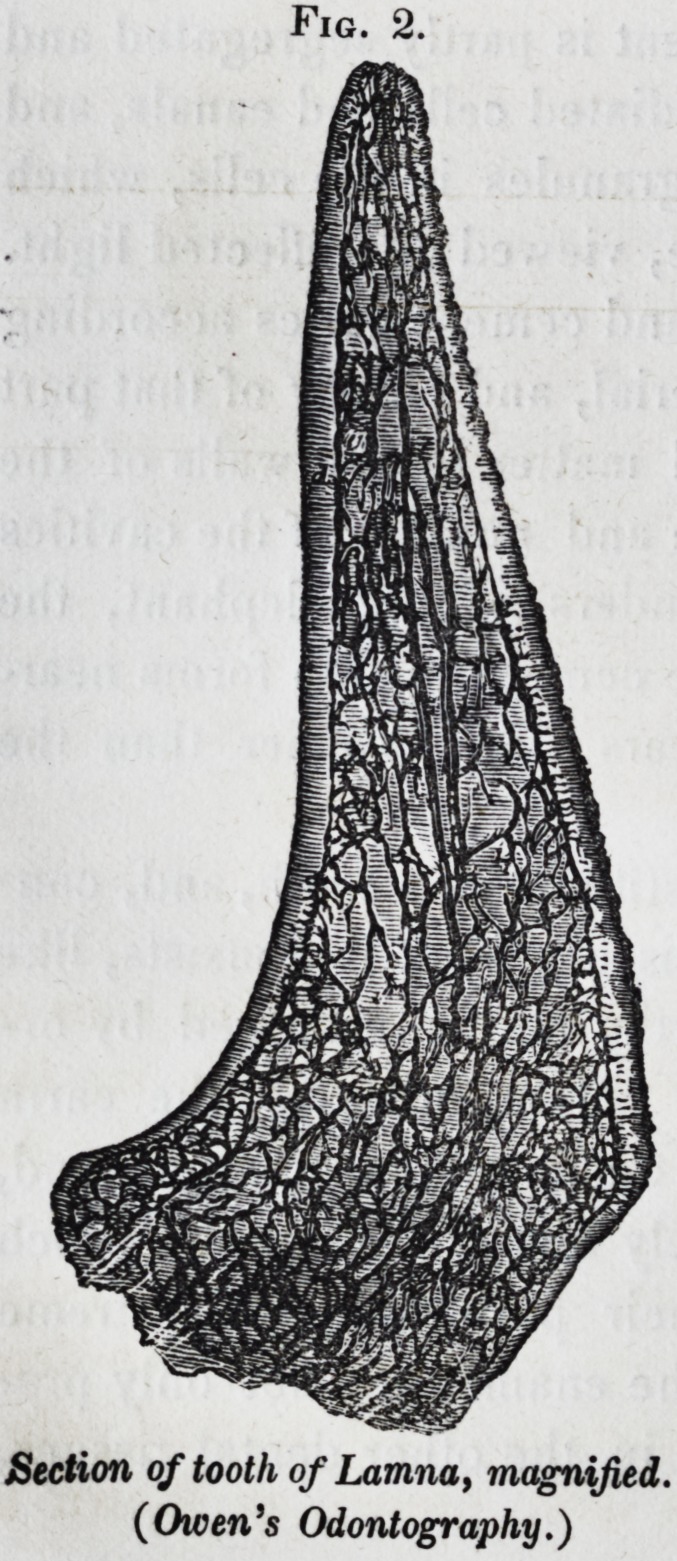


**Fig. 3. f3:**
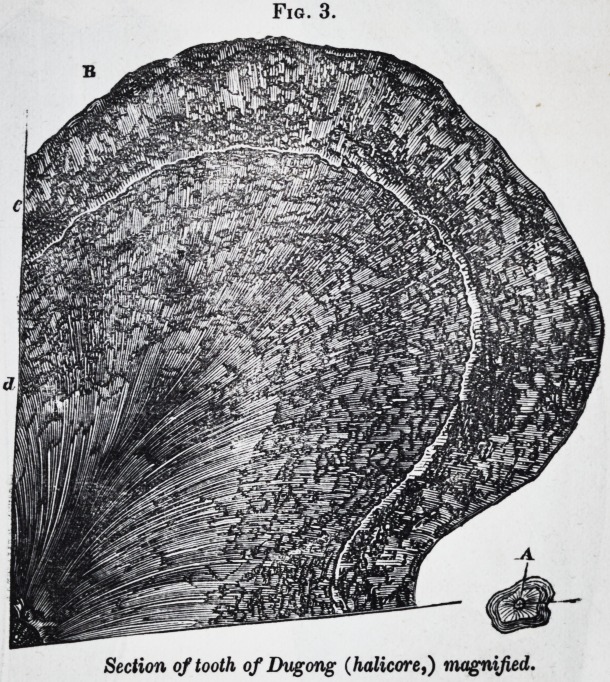


**Fic. 4. f4:**
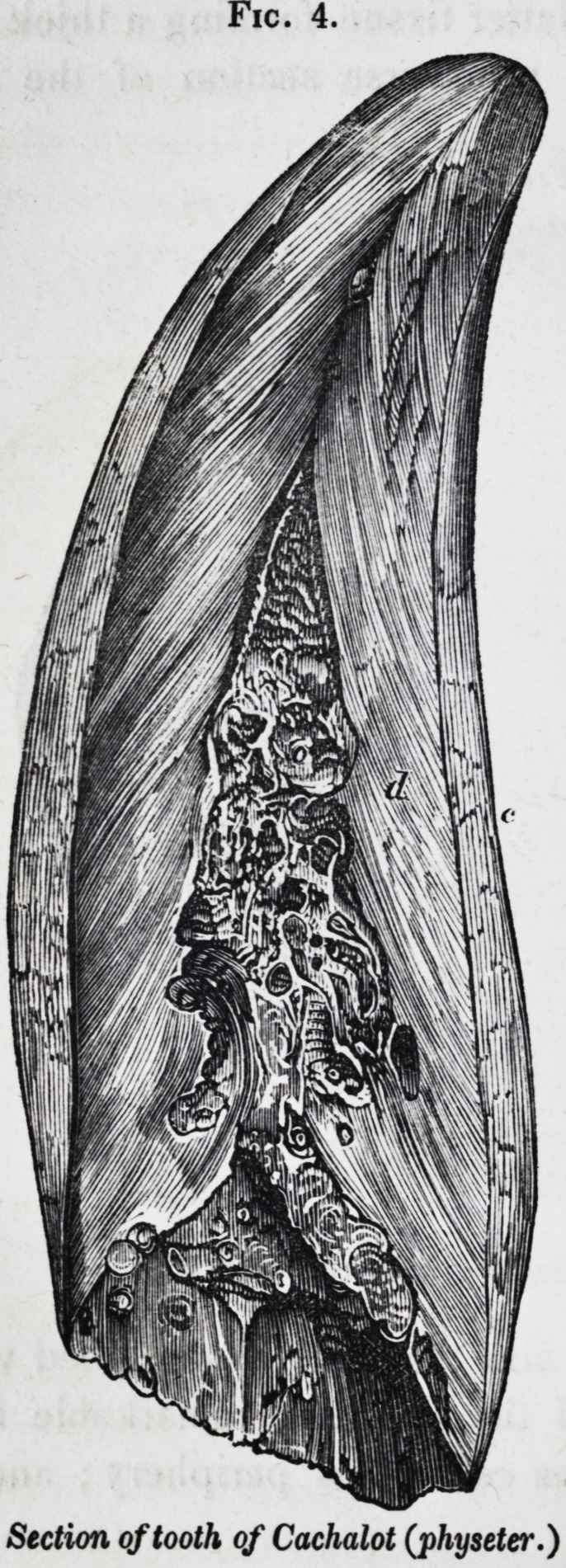


**Fig. 5. f5:**
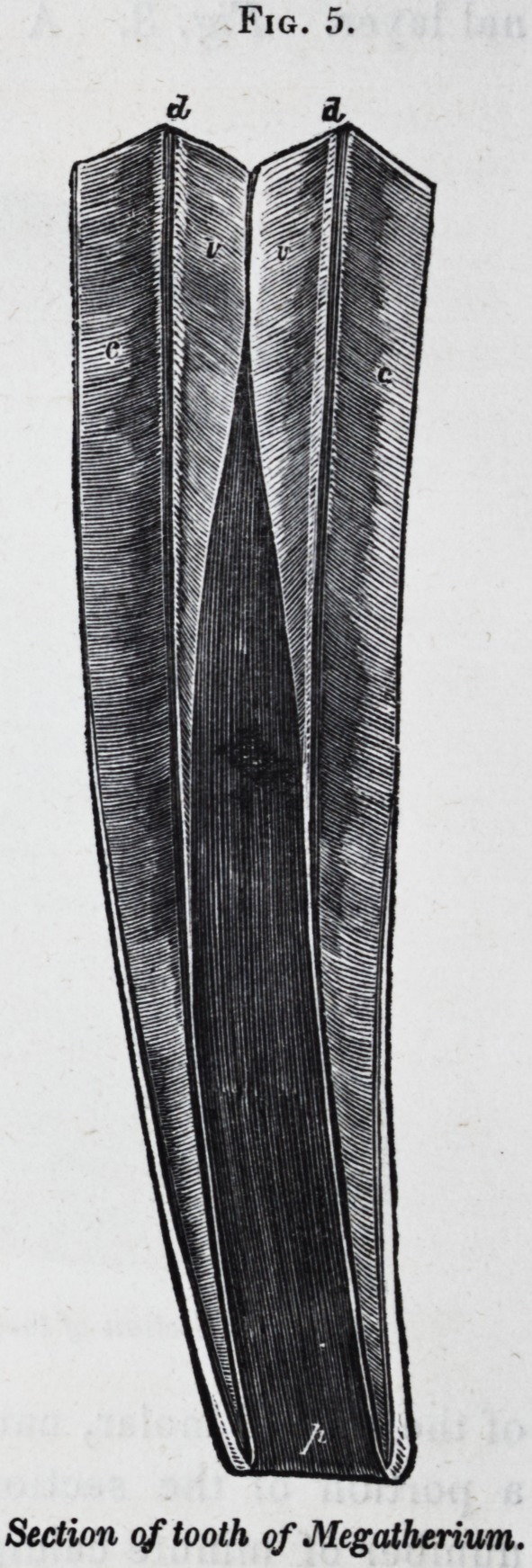


**Fig. 6. f6:**
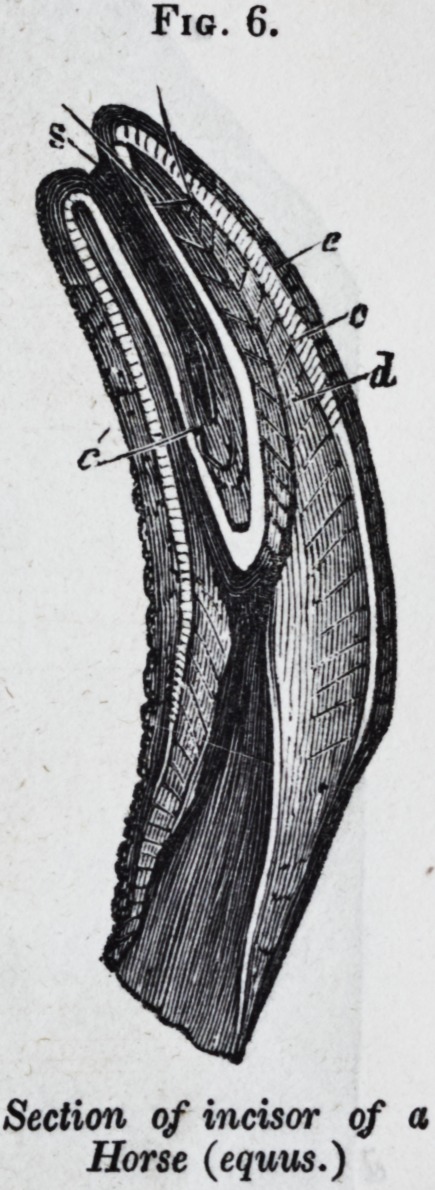


**Fig. 7. f7:**
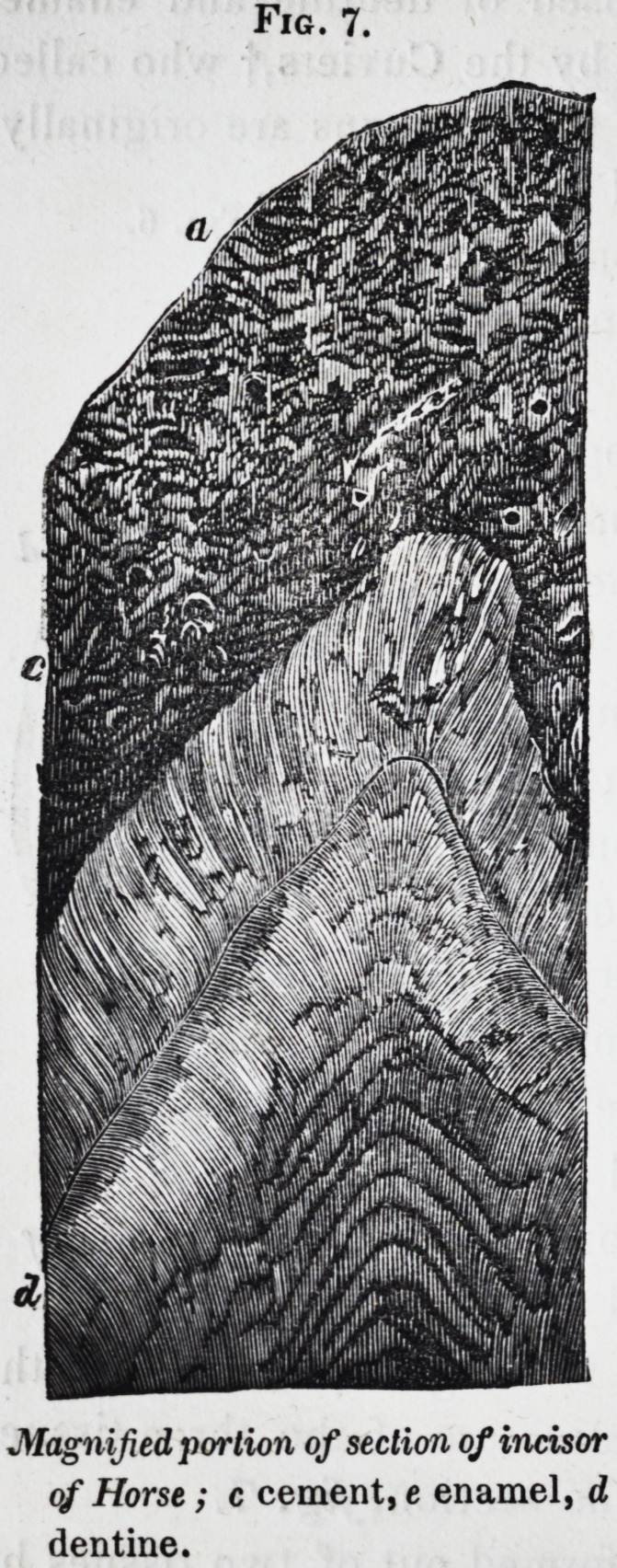


**Fig. 8. f8:**
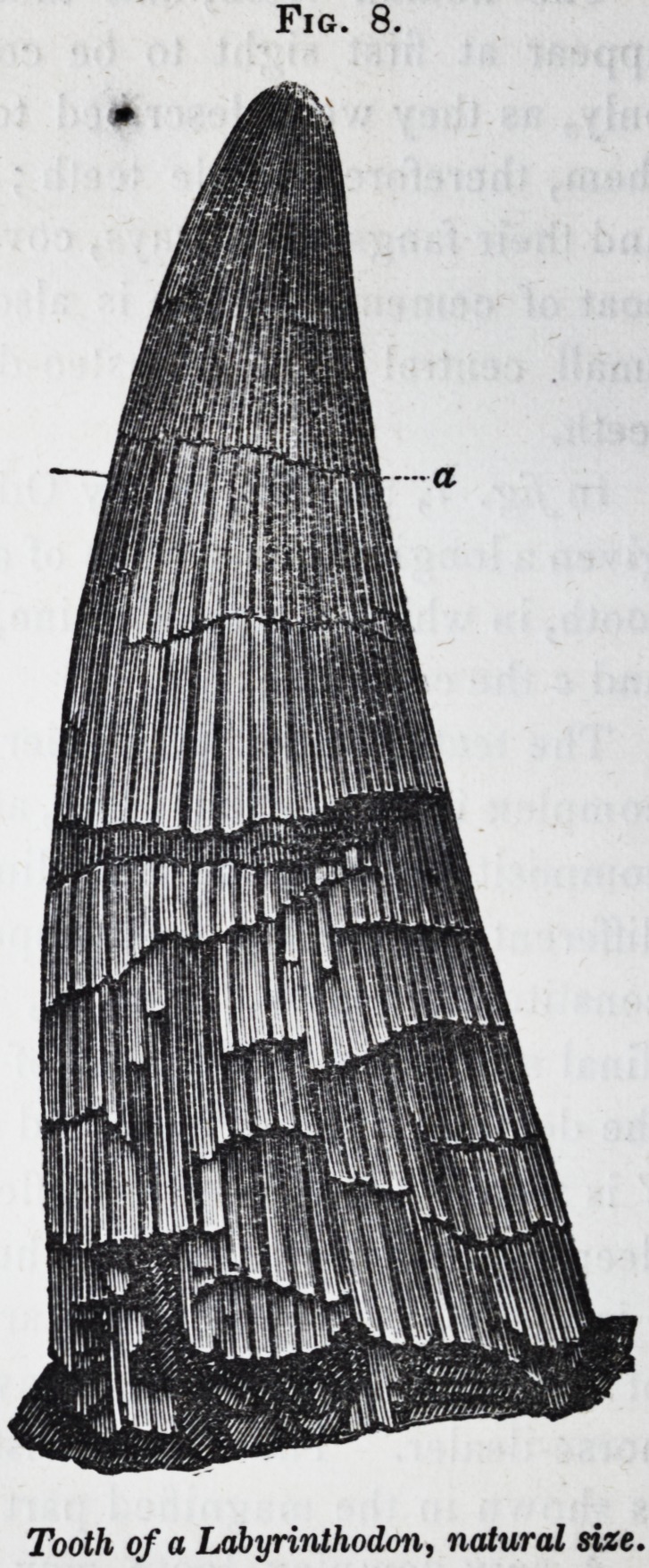


**Fig. 9. f9:**
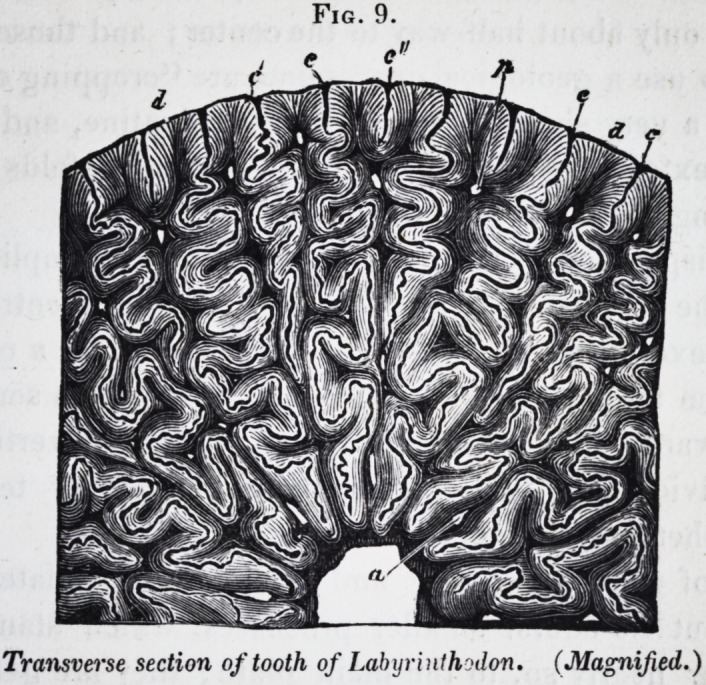


**Fig. 10 f10:**
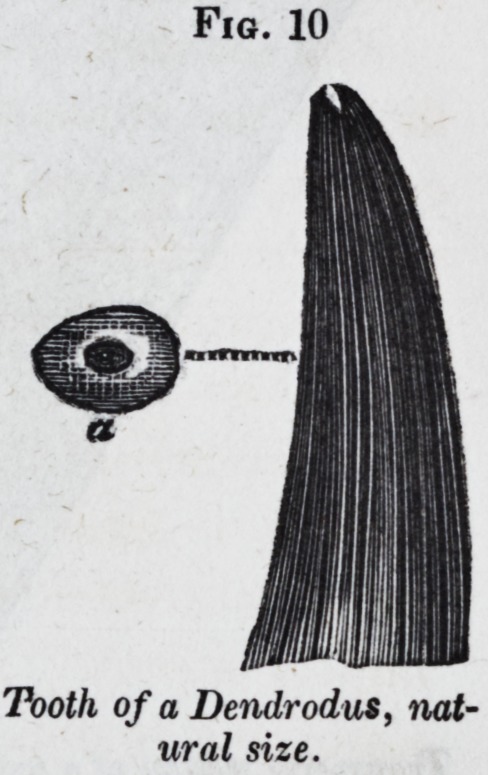


**Fig.. 11 f11:**
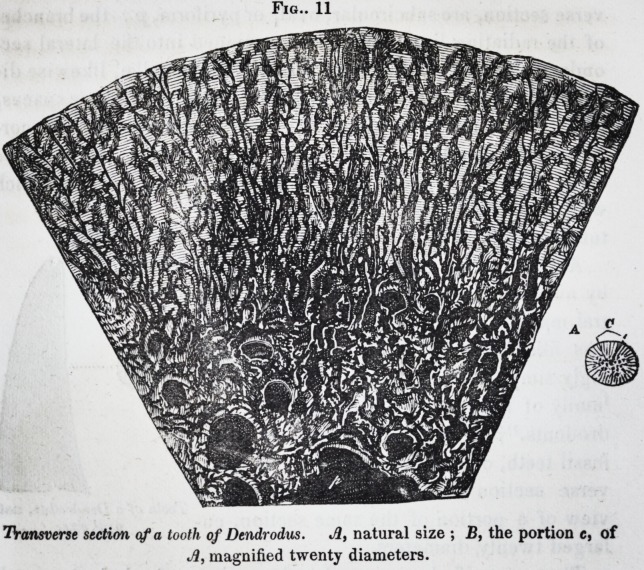


**Fig. 12. f12:**
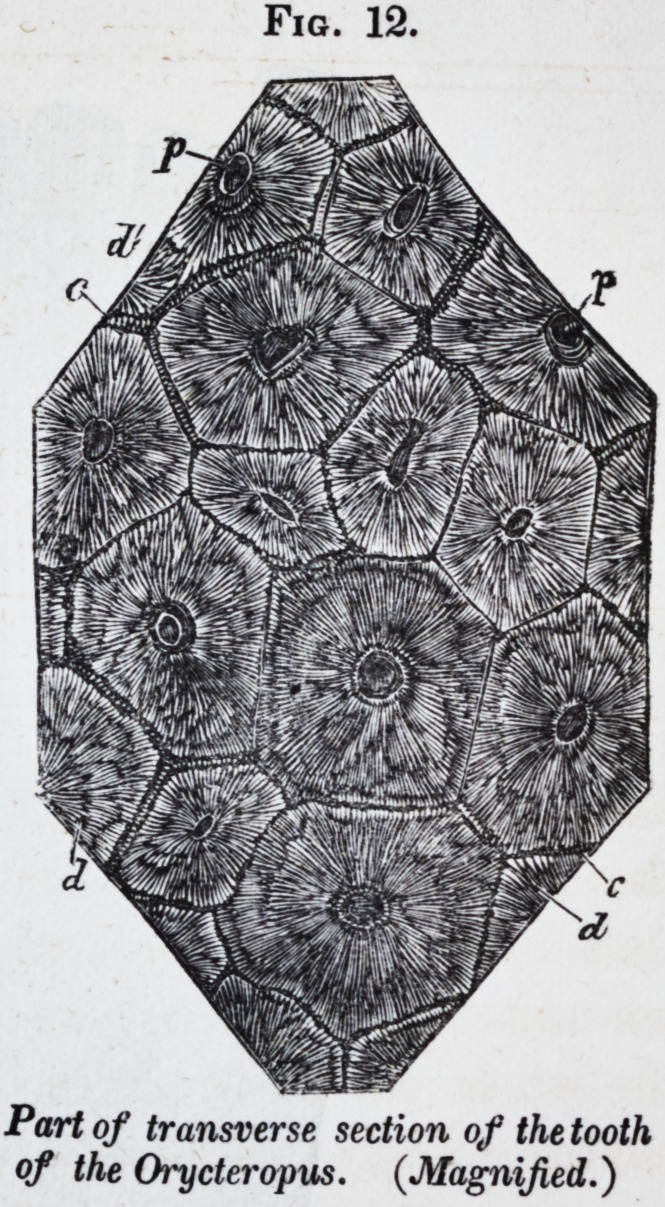


**Fig. 13. f13:**
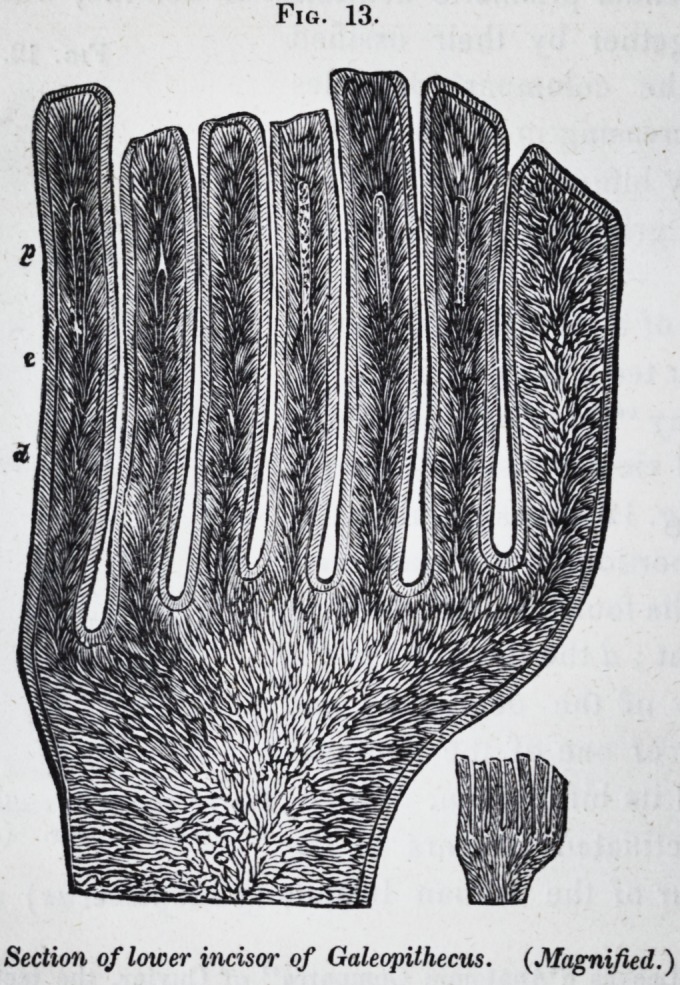


**Fig. 14. f14:**
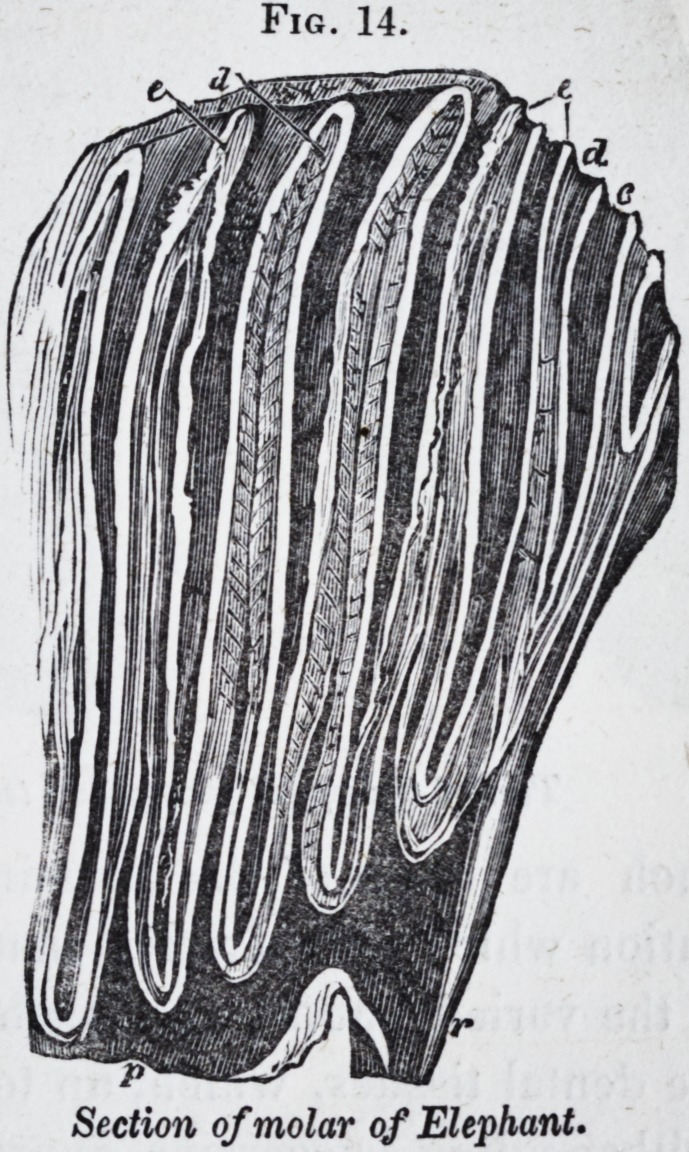


**Fig. 15. f15:**